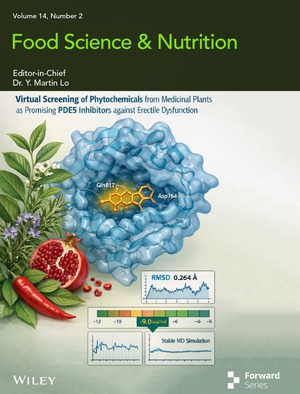# Cover Image

**DOI:** 10.1002/fsn3.71598

**Published:** 2026-03-17

**Authors:** Farouk Boudou, Alaeddine Berkane, Amal Belakredar, Ahcene Keziz, Huda Alsaeedi, Brian A. Murray, Mikhael Bechelany, Ahmed Barhoum

## Abstract

The cover image is based on the article *Virtual Screening of Phytochemicals From Medicinal Plants as Promising PDE5 Inhibitors Against Erectile Dysfunction* by Ahmed Barhoum et al., https://doi.org/10.1002/fsn3.71478.